# Deletion of the Ste20-like kinase SLK in skeletal muscle results in a progressive myopathy and muscle weakness

**DOI:** 10.1186/s13395-016-0119-1

**Published:** 2017-02-02

**Authors:** Benjamin R. Pryce, Khalid N. Al-Zahrani, Sébastien Dufresne, Natalya Belkina, Cédrik Labrèche, Genaro Patino-Lopez, Jérôme Frenette, Stephen Shaw, Luc A. Sabourin

**Affiliations:** 1 Department of Cellular and Molecular Medicine, Faculty of Medicine,, University of Ottawa, Ottawa, ON Canada; 2 Cancer Therapeutics Program, Ottawa Hospital Research Institute, 501 Smyth Rd, BOX 926, Ottawa, ON Canada; 3 Centre de Recherche du Centre Hospitalier Universitaire de Québec-Centre Hospitalier de L’Université Laval, Université Laval, Quebec City, Canada; 4 Département de Réadaptation, Faculté de Médecine, Université Laval, Quebec City, Canada; 5 National Cancer Institute at the National Institutes of Health, Bethesda, MD USA; 6 Laboratorio de Investigación en Inmunología y Proteómica Hospital Infantil de México “Federico Gómez”, México, Distrito Federal México

**Keywords:** Ste20-like kinase, Myofiber stability, Muscle regeneration

## Abstract

**Background:**

The Ste20-like kinase, SLK, plays an important role in cell proliferation and cytoskeletal remodeling. In fibroblasts, SLK has been shown to respond to FAK/Src signaling and regulate focal adhesion turnover through Paxillin phosphorylation. Full-length SLK has also been shown to be essential for embryonic development. In myoblasts, the overexpression of a dominant negative SLK is sufficient to block myoblast fusion.

**Methods:**

In this study, we crossed the Myf5-Cre mouse model with our conditional SLK knockout model to delete SLK in skeletal muscle. A thorough analysis of skeletal muscle tissue was undertaken in order to identify defects in muscle development caused by the lack of SLK. Isometric force analysis was performed on adult knockout mice and compared to age-matched wild-type mice. Furthermore, cardiotoxin injections were performed followed by immunohistochemistry for myogenic markers to assess the efficiency muscle regeneration following SLK deletion.

**Results:**

We show here that early deletion of SLK from the myogenic lineage does not markedly impair skeletal muscle development but delays the regenerative process. Interestingly, adult mice (~6 months) display an increase in the proportion of central nuclei and increased p38 activation. Furthermore, mice as young as 3 months old present with decreased force generation, suggesting that the loss of SLK impairs myofiber stability and function. Assessment of structural components revealed aberrant localization of focal adhesion proteins, such as FAK and paxillin. Our data show that the loss of SLK results in unstable myofibers resulting in a progressive myopathy. Additionally, the loss of SLK resulted in a delay in muscle regeneration following cardiotoxin injections.

**Conclusions:**

Our results show that SLK is dispensable for muscle development and regeneration but is required for myofiber stability and optimal force generation.

**Electronic supplementary material:**

The online version of this article (doi:10.1186/s13395-016-0119-1) contains supplementary material, which is available to authorized users.

## Background

Attachment of myofibers to the extracellular matrix (ECM) is essential for myofiber integrity and muscle function. These attachments are mediated by membrane-associated protein complexes. These complexes also attach to intracellular proteins that mediate myofiber stability through connections to the cytoskeleton. Two distinct protein families mediate attachment to the ECM, the dystrophin-associated glycoprotein complex (DGC) and the integrin/focal adhesion family of proteins [1]. The DGC associates with the ECM through its interaction with the extracellular protein laminin [2, 3]. The loss of several components of the DGC leads to the generation of severe myopathies, the most notable of which is the loss of functional dystrophin, which is the underlying cause of Duchenne muscular dystrophy (DMD) [4]. The alternative attachment system consists of integrin subunits and their associated proteins [1, 5]. Integrin complexes are crucial components of cell-cell and cell-ECM attachments in multiple tissues throughout the body including skeletal muscles [6]. Similar to its binding to the DGC, laminin interacts with the integrin subunits coupling the ECM with the cytoskeleton [7]. Muscle integrin complexes are primarily comprised of heterodimers of α7 and β1 subunits [1]. Muscle-specific deletion of the β1 subunit results in decreased myoblast fusion and severe defects in sarcomere assembly [8]. The loss of functional α7 integrin has been identified as a cause of muscular dystrophy in human patients [9, 10]. Interestingly, α7 integrin has been demonstrated to act as a compensatory mechanism for the loss of dystrophin in DMD animal models [11]. Conversely, ablation of α7 integrin in Mdx mice results in a more severe phenotype than the dystrophin knockout alone and better reproduces the clinical progression of DMD [12]. The integrin complexes also have an essential role in forming the attachment of the tendon and muscle, termed the myotendinous junction (MTJ) [13, 14].

Networks of intracellular complexes form the costamere which attaches membrane proteins to the cytoskeleton [15]. The function of the costamere is to transmit forces laterally from the sarcolemma to the sarcomere as well as to stabilize the intracellular myofiber structure. Downstream components of the integrin signaling system have been found to be vital for the formation of costameres and, similar to the DGC and integrins, loss of proteins within these complexes have a severe impact on muscle integrity. For example, skeletal muscle deletion of integrin associating protein Talin results in decreased force generation as well as a central nuclear myopathy in mice [16–18]. Additionally, focal adhesion kinase (FAK) was found to be essential for the formation of costamere rings and the fusion of myoblasts into multi-nucleated myotubes [19, 20]. Interestingly, downstream components of the integrin signaling system, such as FAK and paxillin, are also upregulated in hypertrophied muscles, suggesting that they are beneficial for muscle growth and function [21].

The Ste20-like kinase (SLK) is a ubiquitously expressed protein found at high levels in developing myogenic and neuronal tissues [22, 23]. Full-length SLK consists of an N-terminal catalytic kinase domain, a coiled-coiled region and a disorganized C-terminal region termed the ATH domain [24, 25]. The ATH region has been shown to bind Ldb1 and LMO4, both of which act to modulate SLK kinase activity [26, 27]. We have shown that SLK is activated downstream of the integrin/FAK/Src signaling system to mediate cellular migration and focal adhesion turnover through paxillin phosphorylation [28, 29]. SLK also functions upstream of RhoA-GTPase to regulate the formation actin stress fibers in smooth muscle cells [30]. Expression of a dominant negative SLK leads to cell cycle arrest, likely due to its ability to control cytoskeletal remodeling at mitotic entry [31, 32]. Supporting this, a role for SLK in microtubule radial organization and Golgi orientation has been demonstrated [33, 34]. Our previous data have shown that SLK is also expressed in mature muscle and co-localizes with Z-band proteins, such as α-actinin. SLK was also found to be preferentially expressed in type I and type IIA fibers in skeletal muscle [23]. In myoblasts, SLK has been demonstrated to be critical for fusion and differentiation [23].

Recently, our analysis of an SLK gene trap model revealed a significant reduction in myosin heavy chain (MyHC) and MyoD+ populations in the developing myogenic compartment, supporting a role for SLK in the development and expansion of embryonic skeletal muscles [35]. However, it is still unclear whether this was due to the observed defects in placental angiogenesis, or as a direct result of SLK deletion within the myogenic lineage. Interestingly, muscle-specific expression of a dominant negative SLK driven off the differentiation-specific human skeletal actin (HSA) promoter resulted in viable mice with enhanced regeneration and myoblast fusion [36].

To investigate the role of SLK during early muscle development and in myoblasts, we performed a muscle specific knockout using the Myf5-Cre knock-in mice and a conditional SLK knockout allele. In contrast to the gene trap model, muscle-specific deletion of SLK results in viable mice with apparently normal myogenic compartments in the embryo. However, adult knockout mice present with a progressive myopathy and impaired muscle function combined with a marked delay in regenerative capacity. Structural analysis of knockout muscle tissues showed a mislocalization of focal adhesion proteins at the periphery of the myofibers. Collectively, these results suggest that SLK is necessary for myofiber integrity and maintenance but is dispensable for complete muscle regeneration and embryonic myogenesis.

## Methods

### Animal care and genotyping

The SLK conditional allele was generated by the insertion of the FRT-flanked Neo cassette downstream of exon 2 with loxP sites flanking exons 2 to 6 of the murine SLK locus. Chimeras were generated on a mixed C57BL/129 background and germline pups in a C57 background were mated with β actin-Flp recombinase transgenic mice to obtain the floxed line (SLK^*fl/+*^). Myf5^*Cre/+*^ mice (Myf5-Cre) were obtained from Jackson Lab [37]. Genotyping was performed using ear clips from 3-week-old weanlings and processed using proteinase K (QIAGEN, Mississauga, ON, Canada). Mice were genotyped based on primers designed flanking the 3′ SLK loxP site. Wild type alleles produced a fragment at 437 bp, whereas the loxP mutant generates a fragment of 471 bp. Cre-positive animals were identified by genotyping for Cre recombinase using sequence specific primers (Table 1). Myf5-Cre mice were crossed to SLK^*fl/fl*^ or SLK^*fl/+*^ mice to obtain heterozygote, wild type and homozygote progenies. Cre-mediated recombination at the SLK locus was assessed by PCR analysis by introducing an additional forward primer upstream of the 5′ loxP site. Non-recombined alleles (>5 kbp) are not amplified.

**Table 1:** Genotyping and recombination PCR primer sequences

5′ loxP forward SLK primer (5′F)	TTGGGGGATGGCTTCGTGCTT
3′ loxP forward SLK primer (3′F)	TGAGGACCTGGGGAGATTGCT
3′ LoxP reverse SLK primer (3′R)	ATGCAGCTGTATCTTCACAAG
5′ Cre primer	GGATTGCTTATAACACCCTGTTACG
3′ Cre primer	TATTCGGATC ATCAGCTACACCAGAG

### Western blotting

For Western blotting, skeletal muscles were frozen in liquid nitrogen and ground with a mortar and pestle. Ground tissues were then lysed in RIPA buffer containing protease and phosphatase inhibitors as previously described [35]. Lysates were then cleared by centrifugation at 14,000RPM for 5 min at 4 °C. Protein concentration was determined by using Bradford protein assay dye reagent (Bio-Rad, Mississauga, ON, Canada). Equal amounts of protein were run on polyacrylamide gels and transferred onto polyvinylidene difluoride membranes (PVDF). Membranes were probed with primary antibodies for 1 h at room temperature, or overnight at 4 °C followed by secondary HRP-conjugated antibodies for 1 h at room temperature. Antibody detection was performed by chemiluminescence and X-ray film exposure.

### Immunofluorescence and immunohistochemistry

Muscles for immunofluorescence were isolated by careful dissection from the tendon, followed by either OCT embedding and snap-freezing in isopentane/liquid nitrogen or formalin-fixed overnight and paraffin embedded. Sections (5 μm) were boiled in 10 mM citrate buffer for antigen retrieval followed by permeabilization for 30 min in 0.3% Triton-X, rinsed in PBS, and incubated in primary antibody overnight. Sections were then incubated for 1 h in secondary antibody and washed in PBS. Slides were mounted in Antifade reagent with DAPI to preserve staining and to identify nuclei (ProLong Gold Antifade). Sections were processed similarly for DAB staining, with an additional incubation in 3% H_2_O_2_ following antigen retrieval, and mounted in Permount mounting media following dehydration.

### Transmission electron microscopy

Muscle specimens were fixed in buffered 2.5% glutaraldehyde for 2 h, rinsed in 50 mM sodium cacodylate buffer, and post-fixed for 90 min in aqueous 2% osmium tetroxide. Following dehydration in an ethanol series and suspension in acetone, specimens were embedded in Spurr’s resin and polymerized at 65 °C overnight. Thin (80 nm) sections were counter-stained with uranyl acetate and lead citrate and observed on a Hitachi H-7100 TEM. Images were captured at the indicated magnifications.

### Cardiotoxin injections

Muscle injury experiments were performed as previously described [36]. Briefly, 10–12-week-old mice were anesthetized with buprenorphine according to Animal Care and Veterinary Services (ACVS) uOttawa guidelines. Tibialis anterior (TA) muscles were injected with 30 μL of 10 μM cardiotoxin (CTX, Sigma-C9759). Contralateral TA muscles were injected with a saline control. Muscles were extracted at 7, 10, and 21 days post injection (DPI). Cross-sectional area and minimum Feret’s diameter were measured on random images of TA muscles stained with H&E (ImageScope; Aperio, Vista, CA, USA).

### Fiber size and central nuclei quantification

For quantification of fiber size, cross sections were stained with hematoxylin and eosin. Cross-sectional areas were calculated using Aperio Imaging software. For soleus muscles, every observable fiber was measured (~50–100/muscle). For TA muscles, fibers from 5 random fields of view were quantified (~300–400 fibers/muscle). Fiber area was then averaged for each muscle taken. Central nuclei were counted from 5 random fields of view on cross sections for each muscle (5/genotype). Total number of fibers with central nuclei from all fields of view was calculated as a percentage of total fibers counted.

### Antibodies

Our rabbit custom polyclonal antibodies for SLK was used throughout this study, as previously described [35]. GAPDH (#5174), P-Y397-FAK (#3283), P-Y118-Paxillin (#2541), P-p38 (#4511), p38 (#9212), P-S9-GSK3β (#9336), GSK3β (#9315), P-SAPK/JNK (#4668), and SAPK/JNK(#9252), were acquired from cell signaling. MHC type 1/slow (M8421), MHC type 2/fast (M4276), γ tubulin (T5192) and Vinculin (V9131) were purchased from Sigma-Aldrich. Antibodies for dystrophin (ab15277) and laminin (ab11575) from Abcam were used for staining. The Myf5 (sc-302) and Myogenin (sc-12732) antibodies were purchased from Santa Cruz. Antibodies for periostin (AF2955) and Pax7 (MAB1675) were from R&D Systems. Finally, total FAK (610088) and total paxillin (612405) were acquired from BD Biosciences.

### Isometric contractile properties and statistical analysis

Mice were first injected with buprenorphine (i.p. 0.1 mg/kg) and then anesthetized with pentobarbital sodium (i.p. 50 mg/kg) 15 min later [38]. The right soleus (Sol) and extensor digitorum longus (EDL) muscles were dissected and incubated in vitro in a buffered physiological salt solution (Krebs-Ringer) supplemented with glucose (2 mg/mL) and a constant bubbling of carbogen (5% CO_2_, 95% O_2_) at 25 °C. After 15 min of equilibration at optimal length (L0), the following contractile properties were measured: time-to-peak twitch tension (TPT, ms) with 0.2 ms square-wave pulses of supramaximal voltage (~25 V) through two platinum electrodes, half-relaxation time (1/2 RT, ms), twitch tension (Pt, g) and maximum tetanic tension (P0, g) for 700 ms at frequencies of 10, 20, 50, 80, 100, and 120 Hz using the Dual-Mode Lever Arm System 305B-LR controlled by the Dynamic Muscle Control and Data Acquisition software (Aurora Scientific Inc. Aurora, Ontario, Canada) [39–41]. At the end of the contractile properties measurements, tendons were removed and muscles were weighed. The cross-sectional areas were estimated by dividing the wet weight by the optimal muscle length multiplied by the muscle density (1.06 g/cm^3^) multiplied by the fiber-to-muscle length ratio for Sol and EDL muscles [42]. All values are expressed as means ± SE. The data were analyzed by two-way ANOVA to determine whether the variations among the experimental groups were significant (InStat software, version 3). When a significant *F* ratio was obtained, a posteriori test was performed (Tukey’s protected least-significant difference test) to determine whether there were any specific differences. The level of significance was set at *p* < 0.05.

### Primary myoblast extraction and in vitro differentiation

Primary myoblast cultures were isolated as previously described [36]. Briefly, hind leg muscles from 6-week-old mice were minced and digested in collagenase/dispase. Cells were collected and grown in Ham’s F-10 media (Sigma-Aldrich, St Louis, MO, USA). Medium was supplemented with 10 ng/mL bFGF. When cultures reached 70%, density medium was switched to 2% horse serum to induce differentiation. Cells were fixed 4 days after medium changed and stained using MF20 or an anti-Myogenin antibody. Fusion index was calculated as previously described [36].

### Fiber isolation and imaging

Primary myofibers were isolated using established methods [43]. Individual EDL muscles were carefully dissected from mice of each genotype and placed in 0.2% collagenase type 1 and incubated at 37 °C. Muscles were periodically triturated in order to release individual fibers. Muscle fibers were removed and fixed in 4% PFA followed by treatment in 0.3% Triton X-100 in order to permeabilize the membranes. Following immunostaining, images of fibers were captured on LSM 510 META confocal microscope.

### Quantitative reverse transcription-polymerase chain reaction

Diaphragm muscle RNA was extracted using Trizol reagent and extraction protocol. RNA was then purified using RNeasy kit (Qiagen). A total of 500 ng of RNA from each sample was used in SuperScript first-strand cDNA synthesis using oligiodT primers (Invitrogen). The indicated targets were amplified using gene-specific primers and SYBR Green Reagent (Bio-Rad). Reactions were carried out on an Applied Bioscience 7500 Fast-Real-Time PCR System. Sequences for α7 integrin, β1 integrin, β1D integrin, and dystrophin are listed in Table 2 [20, 44, 45]. GAPDH was used as a reference sample.

**Table 2:** Quantitative PCR primers

	Forward primer	Reverse primer
α7 integrin	5′-ACTGTCCGAGCCAATATCACCGT-3′	5′-ACCAGTAGTCCCGCCAGCACA-3′
β1 integrin	5′- CATCCCAATTGTAGCAGGCG-3′	5′-CGTGTCCCACTTGGCATTCAT-3′
β1D integrin	5′-CATCCCAATTGTAGCAGGCG-3′	5′-GAGACCAGCTTTACGTCCATAG-3′
Dystrophin	5′-GTGGGAAGAAGTAGAGGACTGTT-3′	5′-AGGTCTAGGAGGCGTTTTCC-3′
GAPDH	5′CATCACCATCTTCCAGGAGCG-R′	5′GAGGGGCCATCCACAGTCTTC-3′

## Results

### Generation of conditional SLK knockout model

**Fig. 1:**
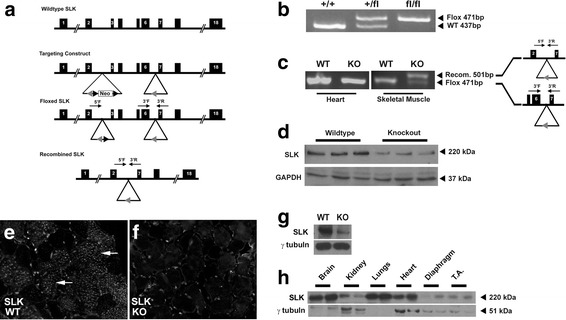
Skeletal muscle deletion of SLK. **a** Schematic representation of the SLK locus and the SLK-targeted allele showing the Frt (*black arrowhead*) and loxP sites (*grey arrowhead*). The location of SLK primers are indicated in the Flp-recombined and Cre-recombined alleles. **b** PCR genotyping of genomic DNA from ear clips for the SLK +/+, SLK +/fl, and SLK fl/fl alleles using 3′F and 3′R primers. **c** Genomic DNA PCR on the heart and skeletal muscle from wild type and knockout mice using the three SLK primers. Recombination product is only observed following Cre-mediated deletion (500 bp). The 3′F and 3′R primers detect non-recombined DNA strand (471 bp). The 5′F and 3′R only generate a product following recombination, whereas the non-recombined allele (>5 Kbp) could not be amplified by the 5′F and 3′R primers. **d** Western blot on adult skeletal muscle lysates from wild type and knockout mice. The membrane was probed with an anti-SLK and anti-GAPDH as a loading control. SLK staining on TA muscles from **e** wild type and **f** knockout mice. Prominent staining in fibers from wild type (indicated with *white arrows*) which is not present in knockout. **g** Western blot analysis of SLK expression in primary cultures of Myf5-Cre:SLK^fl/fl^ myoblasts showing a marked reduction in SLK levels. **h** Total levels of SLK protein within different tissues showing lower expression in normal skeletal muscles

SLK has been shown to be highly expressed in both the myogenic and neuronal compartments of the developing embryo [22, 23]. We have previously demonstrated a role for SLK in muscle development using a transgenic model in which dominant negative SLK (K63R) was expressed in myogenic cells under the control of the human skeletal actin (HSA) promoter [36]. To gain further insights into the role of SLK in muscle development and function, we generated a skeletal muscle-specific deletion of SLK. A conditional SLK allele was generated by flanking exons 3 and 6 of the murine SLK gene with loxP sites (NM_001164639.1) (Fig. 1a). Breeding of SLK^*fl/fl*^ mice to β-actin-Cre resulted in no viable knockout progeny, suggesting that global SLK deletion is embryonic lethal, consistent with our previous findings from a gene trap model (Additional file 1: Table S1) [35].

The muscle-specific SLK knockout was generated by crossing the conditional SLK allele into the Myf5-Cre strain [37]. Myf5 is expressed at 8.5 day post-coitum (dpc) in the rostral somites and between days 10.5 and 12.5 dpc in the developing limb buds, allowing for Cre expression and deletion of SLK within the developing myogenic compartments prior to myofiber formation [46, 47]. In stark contrast to the global SLK knock out models, muscle-specific deletion of SLK resulted in viable progeny at the predicted Mendelian ratios. Interestingly, a small reduction in body mass was observed in younger mice. This difference did not persist beyond 20 weeks of age (Additional file 2: Figure S1A). Otherwise, knockout mice were apparently normal with no obvious anomalies or behavioral changes upon weaning. Presence of the loxP site was confirmed using a forward and reverse primer flanking the 3′ loxP insertion site (Fig. 1b). Muscle-specific deletion of SLK was confirmed by isolating DNA from various skeletal muscle groups, as well as non-myogenic tissue, from adult animals and conducting PCR analysis to assess recombination. As expected, the recombined PCR product was only detected in skeletal muscles from Myf5-Cre:SLK^*fl/fl*^ knockout mice (Fig. 1c). The weak positive signal from the wild type allele is likely due to amplification from contaminating non-muscle DNA as observed for other skeletal muscle-specific knockout studies [8]. Western blot analysis showed a ~80% decrease in total SLK protein levels in skeletal muscle tissues isolated from the knockout mice. This is similar to decrease previously reported for other Myf5-Cre carrying mice, as well as other muscle-specific conditional knockouts (Fig. 1d) [48, 49]. Supporting this, staining for SLK in wild type fibers showed a strong signal within a subset of myofibers that was absent in knockout muscles (Fig. 1e, f). Positive staining was observed between fibers in knockout muscle, likely representing non-myogenic cells expressing SLK [22, 24, 25]. In addition, Western blot analysis of Myf5-Cre:SLK^fl/fl^ primary cultures showed a >90% reduction in total SLK levels, suggesting efficient excision of floxed allele. Interestingly, we observed that SLK levels are relatively low in muscle tissues (Fig. 1g) resulting in higher apparent residual SLK levels. Together, these data suggest that the deletion of SLK is efficient and robust in the skeletal muscles of adult mice.

**Fig. 2:**
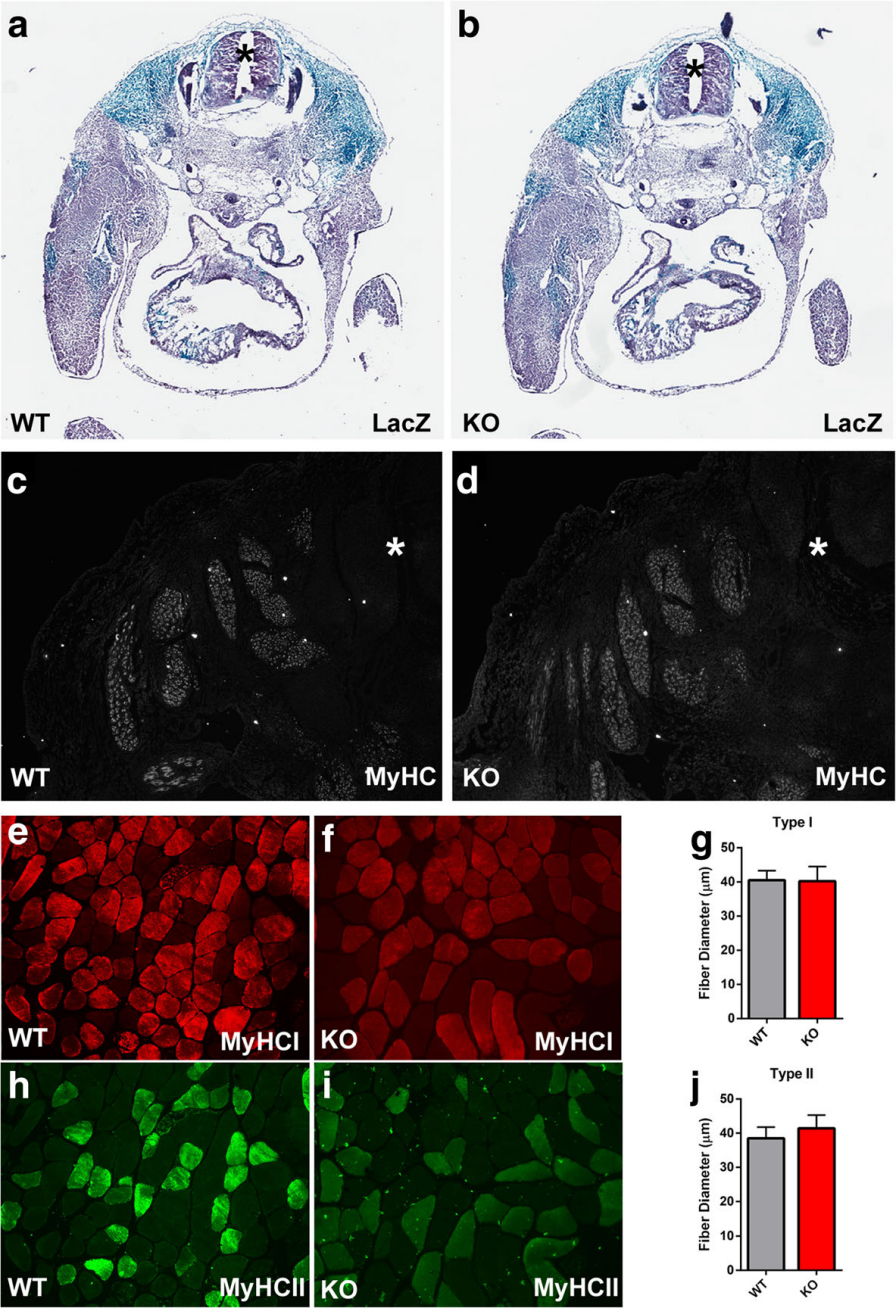
SLK deletion does not affect muscle development. **a**, **b** β-galactosidase histochemistry on cross sections of E12.5 embryos from a wild type (WT) and SLK-null (KO) embryo into the ROSA26R background. The location of neural tube is indicated by an *asterisk*. **c**, **d** MyHC immunofluorescence staining to identify differentiated skeletal muscle tissue within developing E12.5 embryos. The neural tube is indicated by an *asterisk*. **e**, **f** MyHC type I staining on soleus muscles (12 weeks) from wild type (WT) and knockout mice (KO). **g** Minimum Feret’s diameter of type I fibers from each group (*n* = 5/genotype). **h**, **i** MyHC type II staining and **j** Minimum Feret’s diameter of type II fibers (*n* = 5/genotype)

To assess the effect of SLK deletion on muscle development, Myf5-Cre:SLK^*fl/fl*^ mice were crossed with the Rosa26R-LacZ reporter mice and embryos were collected at E12.5. LacZ histochemistry showed that SLK deletion did not affect the spatiotemporal development of the myogenic compartments of the limbs or trunk (Fig. 2a, b). Similarly, staining for MyHC revealed no obvious differences in the distribution of muscle tissue in the embryos at this stage of development (Fig. 2c, d). Together, these results suggest that SLK is dispensable for embryonic myogenesis.

SLK was previously demonstrated to be expressed predominantly within type 1 and type 2A fibers [23]. Surprisingly, analysis of fiber type distribution and size did not differ significantly between 12-week-old wild type and knockout mice (Fig. 2e–j), suggesting that the loss of SLK does not significantly impair postnatal skeletal muscle development or fiber type specification.

### Muscle-specific deletion of SLK leads to myopathy and reduced force generation

**Fig. 3:**
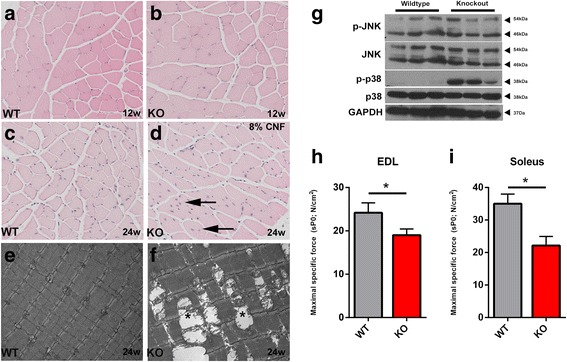
SLK knockout muscles develop central nuclei and decreased force generation. Hematoxylin and eosin staining on 12-week (**a**, **b**) and 24-week (**c**, **d**) animals from each genotype. A high proportion of central nuclei (*arrow*) appeared in numerous sections from SLK-null mice (8% of total nuclei). **e**, **f** Electron photomicrographs from 24-week-old skeletal muscles showing enlarged and degenerating mitochondria (*asterisk*). **g** Western blot analysis of 24-week-old skeletal muscles reveals increased levels of active p38. The Soleus (**h**) and EDL (**i**) muscle fibers were subjected to force measurement assays (7 wild types, 10 knockouts). Maximal specific force was significantly decreased in knockout muscles

To study the effects of SLK deficiency on muscle function and myofiber morphology, we analyzed skeletal muscles from 3- to 6-month-old Myf5-Cre:SLK^*fl/fl*^ knockout mice. Hematoxylin and eosin staining revealed no significant differences between wild type and knockout skeletal muscle in 3-month-old animals (Fig. 3a, b). Surprisingly, the proportion of central nuclei was increased to 8% in 6-month-old knockout mice, while wild type muscles displayed values closer to 1%(Fig. 3c, d). Central nuclei appeared throughout the muscle tissue in different muscle groups, including the TA, EDL, soleus, and diaphragm. These findings suggest that the deletion of SLK may impair the integrity of the myofibers resulting in the activation of a regenerative response in the skeletal muscles of knockout mice. Supporting this, analysis of myofiber diameter at 6 months of age showed a small, but significant, reduction in caliber size in SLK-deficient muscles compared to wild type mice, suggesting the possible onset of an atrophy phenotype following SLK deletion (Additional file 2: Figure S1B).

Further analysis by electron microscopy showed intact sarcomeres in wild type TA muscles, but swollen and degenerating mitochondria were observed sporadically throughout the skeletal muscles of knockout mice (Fig. 3e, f). Interestingly, similar mitochondrial defects were observed in DMPK-/- mice and attributed to muscle degeneration [50]. SLK-null skeletal muscles also presented with increased p38 activity, suggesting the activation of stress response pathways (Fig. 3g). Activation of p38 has also been observed in models of muscular dystrophy, supporting the notion that its activity is due to a defect in myofiber integrity [51]. Mitochondria swelling and degeneration has also been previously associated with constitutive activation of MKK6, a regulator of p38 activity and a marker of increased cellular stress. JNK activity was also assessed, but no obvious increase was observed (Fig. 3g).

Central nuclear myopathies and skeletal muscle stress are commonly associated with a decrease in force generation. Therefore, in order to determine the effect of SLK deletion on muscle physiology, we performed isometric force measurements on isolated EDL and soleus fibers from 3-month-old wild type and knockout mice. As expected, the soleus muscle, composed of primarily type 1 fibers, had a 36.7% reduction in the total force generated (Fig. 3h). The EDL, composed of mostly type 2 fast-twitch fibers, also had a significant decline of 24% in force generation (Fig. 3i). Together, these results strongly support a role for SLK in skeletal muscle function and the integrity of the contractile apparatus.

Consistent damage to myofibers and decreased force generation is often associated with muscle wasting and fibrosis, as is the case in animal models of muscular dystrophy [52]. However, the levels of periostin and active GSK3β, markers of fibrosis and atrophy respectively, did not change significantly between wild type and knockout mice (Additional file 2: Figure S1C). Furthermore, there was no increased deposition of collagen in knockout muscles as detected by Masson’s trichrome staining (Additional file 2: Figure S1D & E).

### Altered localization of focal adhesion proteins in SLK-null skeletal muscle

**Fig. 4:**
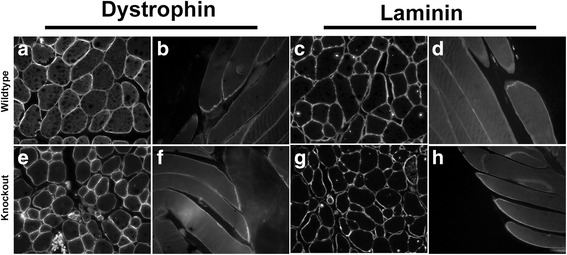
The dystrophin and laminin patterns are unaffected in SLK-null muscles. Dystrophin (**a**, **b**, **e**, **f**) and laminin (**c**, **d**, **g**, **h**) immunostaining on 24-week-old TA muscle from wild type and knockout mice. No apparent differences observed between the groups on either cross or longitudinal sections

SLK has been previously demonstrated to play a role downstream of FAK in mediating cellular migration [28]. Furthermore, direct phosphorylation of paxillin by SLK is essential for efficient focal adhesion turnover and cell motility in fibroblasts [29]. Interestingly, the integrin/FAK signaling axis also plays an essential role in the formation of the costamere and in the maintenance of myofiber integrity [15]. Therefore, to investigate the effect of SLK deletion on costamere or sarcomere morphology, we performed immunostaining on cross sections of skeletal muscles from wild type and knockout mice. Immunofluorescence analysis showed that the distribution of dystrophin and laminin was unaffected by the loss of SLK on skeletal muscle cross sections (Fig. 4a, c, e, g). Similarly, no differences were observed on longitudinal sections, suggesting that the components of the DGC were not affected by the deletion of SLK (Fig. 4b, d, f, h). Total levels of dystrophin also remained unchanged, as observed by Q-PCR (Additional file 3: Figure S2A).

**Fig. 5:**
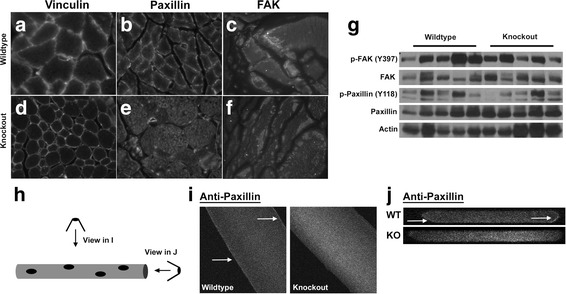
Altered paxillin and FAK distribution in SLK-deficient muscles. **a**, **d** Vinculin immunostaining remained unchanged between wild type and knockout fibers in TA muscles. However, paxillin (**b**, **e**) was readily detectable at the periphery of wild-type myofibers but displayed a cytosolic pattern in knockouts. **c**, **f** FAK localization was also severely disrupted in knockout fibers with no cytoplasmic staining and strong positive clusters underneath the membrane. **g** Western blot analysis for markers of the focal adhesion complex showed no differences in the activation status of either FAK or Paxillin. **h** Illustration of myofibers showing viewpoints of confocal images. **i**, **j** Confocal image of paxillin staining in wild type and knockout fibers

Because SLK was previously shown to play a role in cell migration downstream of FAK/Src signaling, the localization of focal adhesion proteins within the myofibers was also assessed. Similar to dystrophin and laminin, the staining pattern of vinculin was unaffected following SLK deletion (Fig. 5a, d). Interestingly, paxillin localization was predominantly found within the myofibers of SLK-deficient skeletal muscle, whereas wild type controls showed distinct peripheral staining (Fig. 5b, e). Additionally, FAK localization was altered in a similar pattern with a “patchy” distribution throughout the myofibers (Fig. 5c, f). Further analysis of paxillin distribution by confocal microscopy on isolated myofibers showed a similar mislocalization pattern in knockout fibers (Fig. 5h–j). Focal adhesion components have been shown to be critical for myotendinous junction function [17, 18, 48]. The mislocalization of paxillin and FAK in SLK-deficient muscles strongly suggests a defect in the organization of the myotendinous junction, a critical mediator of myofiber stabilization. Activation status of both FAK and paxillin was highly variable in both wild type and knockout muscles. However, quantitation and normalization to total FAK or paxillin did show any differences between the genotypes (data not shown and Fig. 5g). Taken together, our data suggest that the myopathy observed in the SLK-deficient muscles is due in part to underlying defects in the costamere or sarcomere organization due to altered localization of components of the focal adhesion complex. As loss of α7β1 integrins show similar muscle phenotypes as SLK deficiency [8–10], we assessed the levels of these integrins in muscles from both genotypes by Q-PCR. However, no changes were observed between wild type and knockout mice (Additional file 3: Figure S2B–D).

### Muscle regeneration is delayed in SLK-null myofibers

**Fig. 6:**
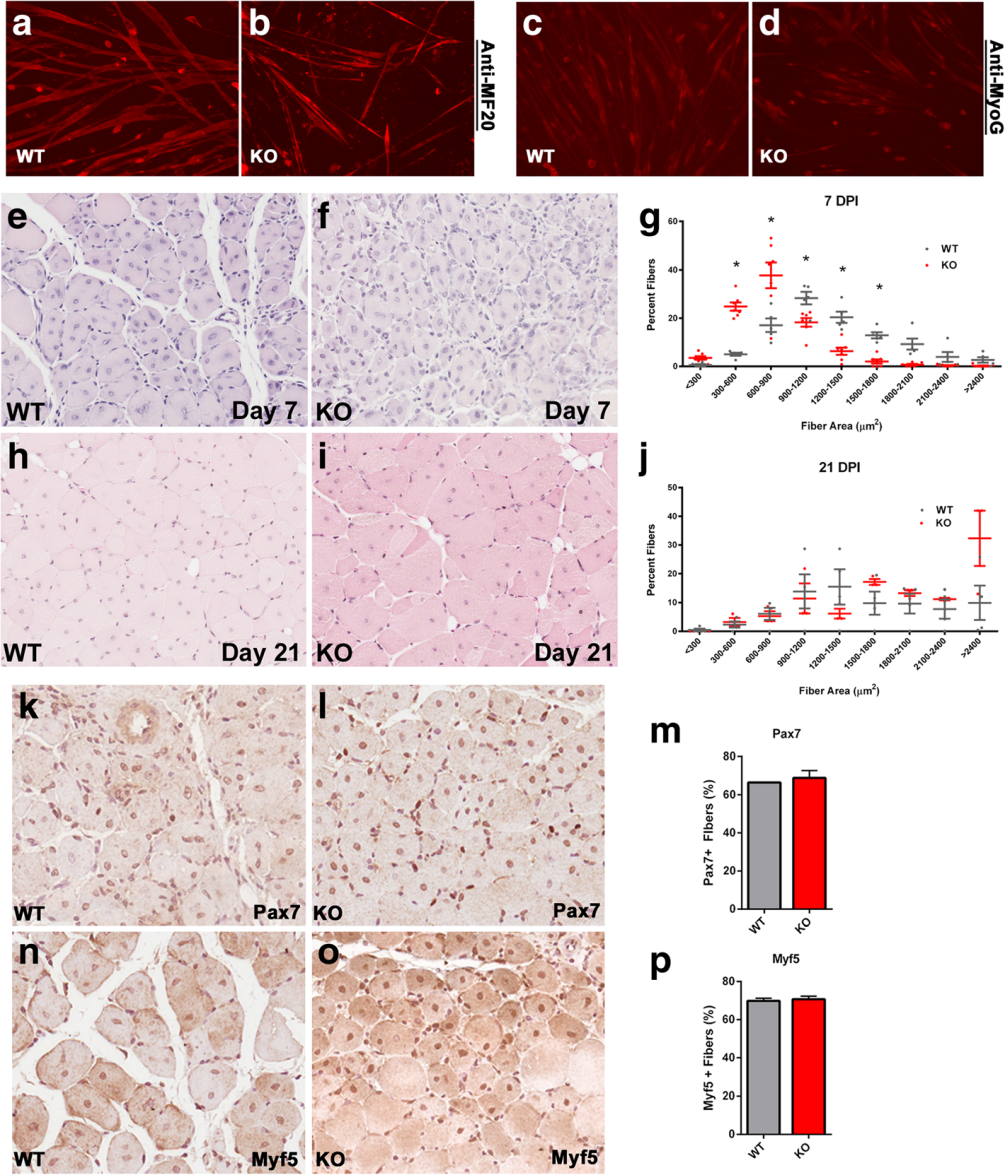
SLK-deficient muscles display a delayed regenerative response. Primary myoblasts isolated from SLK^fl/fl^ mice were treated with AdLacz or AdCre and then differentiated. After 4 days, the cultures were stained with MF-20 (**a**, **b**) or Myogenin (**c**, **d**) and myoblast fusion was assessed (AdLacZ 45.1%, AdCre 10.7%, *p* < 0.01). **e**–**j** The TA muscles of 10–12-week-old mice were injected with cardiotoxin and muscle regeneration was assessed by measuring fiber area. SLK-deficient mice showed a reduction in caliber size 7 days post-injection that was no longer apparent at 21 days, suggesting a delayed response (*n* = 5/genotype). Seven-day regenerates were also stained for Pax7 (**k**–**m**) and Myf5 (**m**–**p**). No changes in Pax7+ or Myf5+ nuclei were observed between the groups (*n* = 3/genotype)

Previously, we reported that the expression of a truncated dominant negative SLK construct reduced the fusion capacity of C2C12 myoblasts [23]. Therefore, we initially assessed the differentiation potential of SLK-deficient myoblasts. Primary myoblast cultures were derived from SLK^fl/fl^ mice and infected with Ad-LacZ control adenovirus or Ad-Cre in order to delete SLK. Compared to Ad-LacZ infected controls, SLK-deleted myoblasts had a lower fusion capacity, reduced from 60% in the wild type to 15% in the knockout, but still retained the expression of differentiation markers such as MyHC and MyoG (Fig. 6a–d).

Muscle regeneration proceeds through the activation and proliferation of satellite cells that acquire Myf5 expression rapidly following their activation [43]. To investigate the role of SLK in muscle repair, we performed cardiotoxin injections into the TA muscle of wild type and knockout mice. Muscle sections were collected at 7 and 21 days post injection (DPI), and the cross-sectional area of the myofibers was measured. Regenerating SLK-deficient muscles displayed a decreased fiber size at 7 DPI compared to knockout animals (Fig. 6e–g). However, fiber sizes of knockout mice where comparable to those of wild type muscles by 21 DPI, suggesting that the loss of SLK delays but does not completely block the regenerative process (Fig. 6h–j). Analysis of the myogenic lineages in regenerating muscles revealed that the number of Myf5+ and Pax7+ cells were similar in wild type and knockout muscles, suggesting that the activation and expansion of satellite cells proceeded normally in SLK-null muscles (Fig. 6k–p). Together, these data show that the loss of SLK impairs myoblast fusion in vitro but only results in a delay in regeneration without any apparent long-term regenerative defects in vivo.

## Discussion

Our data show that mice lacking SLK in skeletal muscles are viable and fertile but present with a progressive central nuclear myopathy and muscle weakness that is readily apparent as early as 3 months of age. In addition, we show that SLK plays a role in the structural integrity of the myofibers and is necessary for maximal force generation. Deletion of SLK from the myofibers resulted in altered localization of proteins of the focal adhesion complex, likely contributing to myofiber instability. Overall, these observations suggest that SLK is dispensable for muscle development and regeneration but is critical to the integrity and the structural organization of the myofibers.

Previously, we have shown that the expression of a dominant negative SLK construct in C2C12 myoblasts blocked fusion and inhibited terminal differentiation. Consistent with this, primary myoblasts deficient in SLK also had a significant reduction in their fusion index. Surprisingly, the deletion of SLK in myoblasts in vivo did not result in a severe impairment of muscle regeneration. Furthermore, SLK-deficient muscles appear to form normally during embryonic myogenesis, indicating that myoblast fusion in embryonic myoblasts is unaffected by SLK deletion. These findings are similar to what has been observed for PKC-θ [53]. Indeed, PKC-θ has been shown to regulate myoblast fusion through upregulation of β-integrin D and caveolin-3 by a FAK-dependent mechanism. The PKC-θ-null mouse is viable, suggesting that myoblasts may activate a compensatory mechanism in vivo, allowing for efficient embryonic development and muscle repair. Similar to the deletion of SLK, PKC-θ-null mice had a reduced body mass upon weaning and presented with a mild muscle wasting phenotype, suggesting that PKC-θ is required for proper muscle development and stability. One possibility is that critical differences in extracellular stimuli are likely to prevent the activation of these compensatory pathways in vitro. Similarly, the deletion of FAK in culture prevented myoblast fusion in vitro, but only caused a delay in muscle repair in vivo [20]. The in vivo and in vitro differences shared between the SLK, FAK, and PKC-θ-null mice highlight the existence of important compensatory pathways that cannot be activated in vitro.

The molecular mechanisms of SLK signaling have been predominantly studied in fibroblast cell lines. In these cells, a role for SLK at the leading edge of migrating cells has been established. SLK has been shown to be activated downstream of FAK and Src signaling and to play a role in cell migration following scratch wounding in vitro. In addition, SLK has been demonstrated to phosphorylate the adapter paxillin to induce focal adhesion turnover, a process necessary for cell motility. This is of particular importance in developing myofibers as the components of the focal adhesion complex play a pivotal role not only in myoblast fusion but also in the development and integrity of the costameres and attachment at the MTJ. Costameres are active sites for signaling and sarcomere attachment to the ECM. Deletion or mutations in costamere-associated proteins result in an increase in central nuclei, decreased force generation and, in some cases, lethality. Our studies show that SLK-deficient muscles display altered localization of FAK and paxillin, two important costamere proteins. Interestingly, vinculin localization was not affected, suggesting that SLK deletion might affect a subset of costameric proteins. Nevertheless, this is likely to affect costameric signaling and to result in weaker attachment of the MTJ and the sarcomere. Based on our findings, SLK appears to be a regulator of myofiber integrity and stability. However, the phenotype observed the following SLK deletion was much less severe than for other cytoskeletal regulators such as Talin and ILK [17, 18, 48, 54]. This may be in part due to compensation from an alternative pathway that remains to be uncovered. Nevertheless, SLK deficiency results in similar mislocalization of focal adhesion proteins within the myofiber.

We have previously reported that muscle-specific overexpression of a dominant negative SLK from the human skeletal actin promoter (HSA-SLK^K63R^) results in enhanced muscle regeneration as well as an increase in the fusion index of primary myoblasts [36]. Furthermore, expression of HSA-SLK^K63R^ also affected muscle organization during development with a marked reduction in litter sizes. Since Pax7+ satellite cells arise from embryonic myoblasts, most of which express Myf5 during development, the Myf5-Cre model would mediate deletion of SLK during early embryogenesis and in adult satellite cells. Surprisingly, deletion of SLK had no apparent effect on embryonic development but caused a delay in muscle regeneration following cardiotoxin induce injury. However, SLK deletion significantly reduced primary myoblast fusion in vitro. One likely possibility is that SLK plays kinase-dependent and -independent roles during the differentiation and the maturation process. The expression of a full-length kinase dead SLK in differentiating cells would still allow its scaffolding functions in the HSA-K63R model. The activation of kinase-dependent pathways would be impaired, leading to the developmental delay previously observed [36]. However, in the SLK-null model, both kinase-dependent and independent processes would be impaired during development and repair. The loss of scaffolding functions in the SLK-null muscles likely leads to anchoring defects of focal adhesion proteins, as these were not observed in the HSA-K63R model. These findings may also explain why known downstream targets of SLK, such as JNK-1 and RhoA, remained unchanged in knockout skeletal muscle (data not shown). The observed differences in regenerative phenotype between the two models could also be due in part to the use of distinct genetic promoters. The Myf5 gene is highly expressed following the activation of satellite cells and remains high in proliferating myoblasts. Conversely, the HSA promoter is active upon cell cycle withdrawal. This supports a role for SLK during myoblast proliferation and fusion and would suggest that its activity needs to be downregulated in order to facilitate myoblast differentiation. Employing the use of the HSA-Cre and the conditional SLK knockout allele may be useful in order to reconcile these phenotypes.

We have previously shown a role for SLK in cell cycle progression through G2/M [31]. Interestingly, we have also observed a decrease in SLK activity prior to myoblast fusion, suggesting that downregulation of SLK activity may be necessary for cell cycle exit and differentiation [23]. The observed regenerative phenotype observed in our knockout mice may reflect a short delay in the expansion of the myoblast population following satellite cell activation, as SLK has been demonstrated to be a mediator of cellular proliferation.

We and others have uncovered a number of substrates for SLK. Phosphorylation of paxillin by SLK was shown to be critical for optimal migration of mouse embryonic fibroblasts [29]. Additionally, RhoA has been identified as an SLK target in smooth muscle cells [30]. ASK1 was shown to be regulated by SLK, providing a link between SLK activity and p38/JNK-1 activation [55]. However, assessment of these pathways did not yield any insight as to the molecular mechanisms by which SLK controls myofiber integrity. Assessments of p38 activity in our model conflicted with previously published data in which SLK phosphorylation of ASK1 was postulated to activate ASK1, resulting in subsequent activation of p38. However, p38 activity was drastically increased in SLK-null skeletal muscle, suggesting alternative SLK-independent regulation of this pathway in skeletal muscle. Alternatively, increased p38 activity could reflect the activation of a stress response induced by the loss of myofiber integrity. The identification of muscle-specific substrates for SLK will undoubtedly provide insights into the role of this kinase in developing muscles and mature myofibers.

## Conclusions

Overall, our data show that deletion of SLK in myogenic precursors did not overtly affect muscle development but induced a mild myopathy that was readily apparent in older mice. Activation of the stress response pathways and decreased force generation suggest that the deletion of SLK impairs myofiber stability. This decreased functionality is due in part to mislocalization of adhesion proteins at the MTJ. These results suggest that SLK is dispensable for muscle regeneration and development but is required for the stability of myotendinous junctions and overall muscle integrity.

## Additional files

Additional file 1: Table S1. β-actin Cre × SLK^fl/fl^ offspring genotype. (DOC 27 kb)
Additional file 2: Figure S1. SLK knockout mice display reduced body mass, muscle size, but show no increase in fibrosis. (A) Body mass of wild type and knockout littermates was measured from 4 weeks to 22 weeks (*n* = 5/genotype). (B) Fiber cross-sectional area was measured in wild type and knockout mice at 3 and 24 weeks (*n* = 5/genotype). (C) Western blot for markers of fibrosis and atrophy on 6-month-old skeletal muscle lysates. (D, E) Masson’s trichome staining on 6-month-old skeletal muscle cross sections from wild type and knockout muscle. (TIF 5209 kb)
Additional file 3: Figure S2. Q-PCR analysis for structural proteins. RNA was extracted from 6-month-old mice (5/genotype). Five hundred nanogram of RNA template was used for cDNA synthesis. RT-PCR for (A) β1integrin, (B) β1D integrin, (C) dystrophin and (D) α7integrin was performed using gene-specific primers. (TIF 1186 kb)

## Abbreviations

ACVS: Animal care and veterinary services
ANOVA: Analysis of variance
bFGF: Basic fibroblast growth factor
DAB: 3,3′-Diaminobenzidine
DAPI: 4′,6-diamidino-2-phenylindole
DGC: Dystrophin-associated glycoprotein complex
DMD: Duchenne muscular dystrophy
dpc: Days post coitum
DPI: Days post injection
ECM: Extracellular matrix
EDL: Extensor digitorum longus
FAK: Focal adhesion kinase
GSK3β: Glycogen synthase kinase 3β
H&E: Hematoxylin and eosin
HSA: Human skeletal actin
ILK: Integrin-linked kinase
MTJ: Myotendinous junction
MyHC: Myosin heavy chain
PAGE: Polyacrylamide gel electrophoresis
PBS: Phosphate-buffered saline
PKC: Protein kinase C
PVDF: Polyvinylidene difluoride membranes
RIPA: Radio-immunoprecipitation assay
SDS: Sodium dodecyl sulfate
SE: Standard error
SLK: Ste20-like kinase
Sol: Soleus
TA: Tibialis anterior
TEM: Transmission electron microscope
