# Supplementary material for: Deletion of the Ste20-like kinase SLK in skeletal muscle results in a progressive myopathy and muscle weakness
Source: Skelet Muscle. 2017 Feb 2;7:3. doi: 10.1186/s13395-016-0119-1 (PMC5288853; doi:10.1186/s13395-016-0119-1)
Supplement: Additional file 1: Table S1. — β-actin Cre × SLKfl/fl offspring genotype. (DOC 27 kb) [file 13395_2016_119_MOESM1_ESM.doc]

**Supplemental Table 1: β-actin Cre x SLK^fl/fl^** Offspring Genotype

|  | **Actual** | **Expected** |
| --- | --- | --- |
| **Heterozygote (SLK +/fl)** | 80/116 (69%) | 58/116 (50%) |
| **Wild Type (SLK +/+)** | 36/116 (31%) | 29/116 (25%) |
| **Knockout (SLK fl/fl)** | 0/116 (0%) | 29/116 (25%) |
