# Supplementary figures and images for: Deletion of the Ste20-like kinase SLK in skeletal muscle results in a progressive myopathy and muscle weakness
Source: Skelet Muscle. 2017 Feb 2;7:3. doi: 10.1186/s13395-016-0119-1 (PMC5288853; doi:10.1186/s13395-016-0119-1)

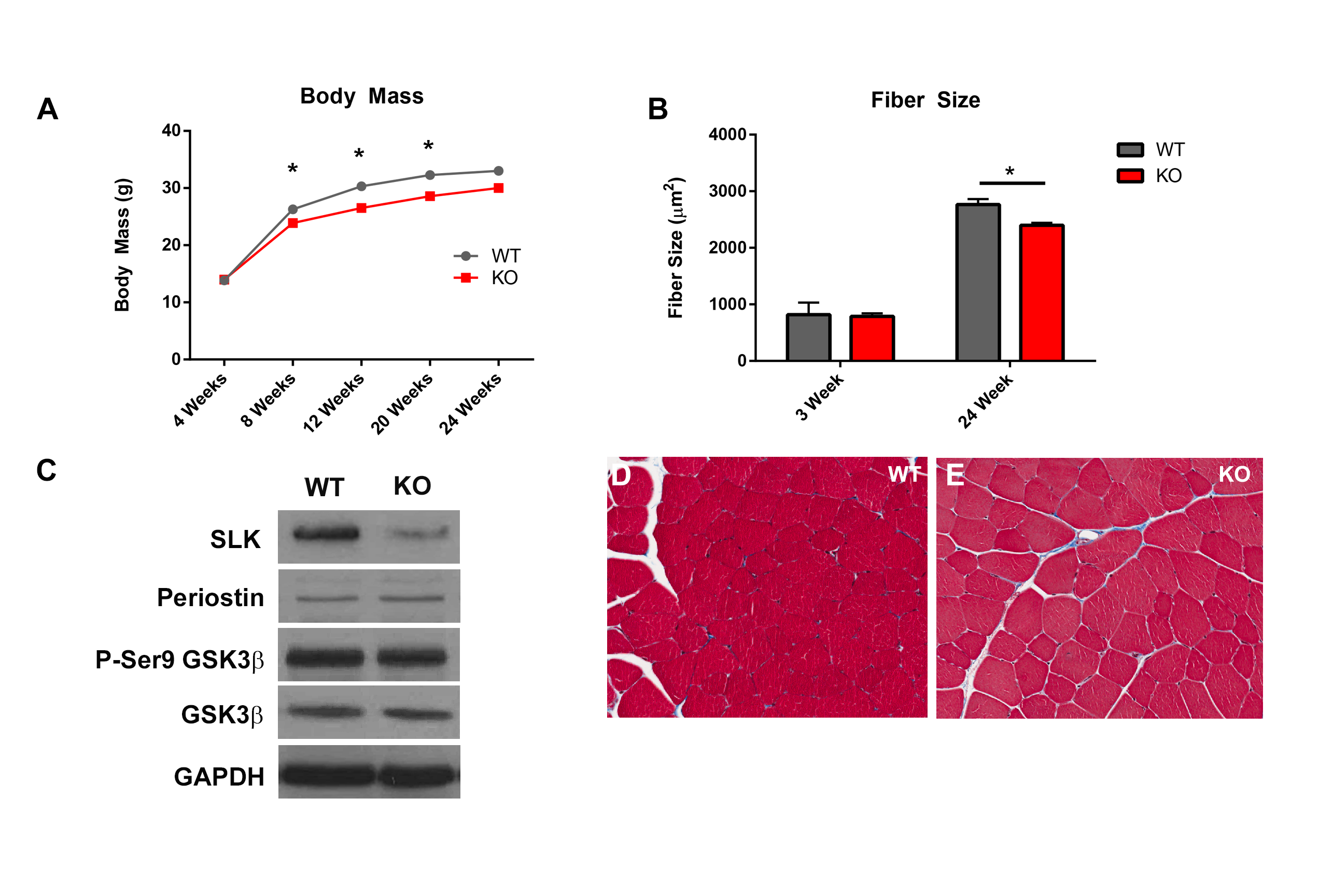

Supplement: Additional file 2: Figure S1. — SLK knockout mice display reduced body mass, muscle size, but show no increase in fibrosis. (A) Body mass of wild type and knockout littermates was measured from 4 weeks to 22 weeks (n = 5/genotype). (B) Fiber cross-sectional area was measured in wild type and knockout mice at 3 and 24 weeks (n = 5/genotype). (C) Western blot for markers of fibrosis and atrophy on 6-month-old skeletal muscle lysates. (D, E) Masson’s trichome staining on 6-month-old skeletal muscle cross sections from wild type and knockout muscle. (TIF 5209 kb) [file 13395_2016_119_MOESM2_ESM.tif]

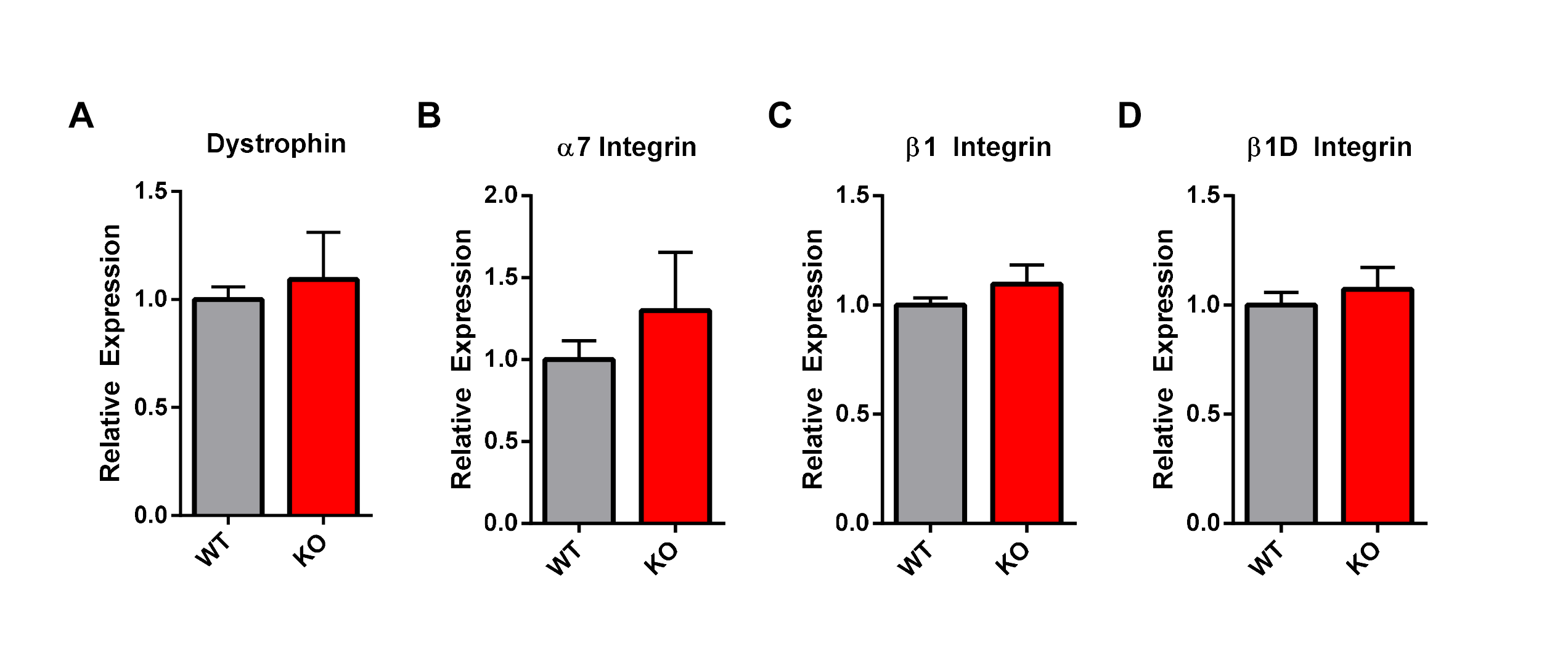

Supplement: Additional file 3: Figure S2. — Q-PCR analysis for structural proteins. RNA was extracted from 6-month-old mice (5/genotype). Five hundred nanogram of RNA template was used for cDNA synthesis. RT-PCR for (A) β1integrin, (B) β1D integrin, (C) dystrophin and (D) α7integrin was performed using gene-specific primers. (TIF 1186 kb) [file 13395_2016_119_MOESM3_ESM.tif]
